# Case Report: Early detection of pancreatic pre-cancer lesion in multimodal approach with exosome liquid biopsy

**DOI:** 10.3389/fonc.2023.1170513

**Published:** 2023-05-11

**Authors:** Harmeet Dhani, Juan Pablo Hinestrosa, Jesus Izaguirre-Carbonell, Heath I. Balcer, Razelle Kurzrock, Paul R. Billings

**Affiliations:** ^1^ Biological Dynamics, Inc., San Diego, CA, United States; ^2^ Georgetown University, Washington, DC, United States; ^3^ Medical College of Wisconsin, Milwaukee, WI, United States; ^4^ Worldwide Innovative Network (WIN) Consortium for Precision Medicine, Villejuif, France

**Keywords:** exosomes, early cancer detection, pancreatic ductal adenocarcinoma, IPMN, extracellular vesicles, early diagnosis, clinical utility

## Abstract

**Background:**

The detection of pancreatic ductal adenocarcinoma (PDAC) lesions at pre-cancerous or early-stages is critical to improving patient survival. We have developed a liquid biopsy test (ExoVita^®^) based on the measurement of protein biomarkers in cancer-derived exosomes. The high sensitivity and specificity of the test for early-stage PDAC has the potential to improve a patient’s diagnostic journey in hopes to impact patient outcomes.

**Methods:**

Exosome isolation was performed using alternating current electric (ACE) field applied to the patient plasma sample. Following a wash to eliminate unbound particles, the exosomes were eluted from the cartridge. A downstream multiplex immunoassay was performed to measure proteins of interest on the exosomes, and a proprietary algorithm provided a score for probability of PDAC.

**Results:**

We describe the case of a 60-year-old healthy non-Hispanic white male with acute pancreatitis who underwent numerous invasive diagnostic procedures that failed to detect radiographic evidence of pancreatic lesions. Following the results of our exosome-based liquid biopsy test showing "High Likelihood of PDAC", in addition to KRAS and TP53 mutations, the patient decided to undergo a robotic pancreaticoduodenectomy (Whipple) procedure. Surgical pathology confirmed the diagnosis of high-grade intraductal papillary mucinous neoplasm (IPMN), which was consistent with the results of our ExoVita^®^ test. The patient’s post-operative course was unremarkable. At five-month follow-up, the patient continued to recover well without complications, in addition to a repeat ExoVita test which demonstrated “Low Likelihood of PDAC”.

**Conclusion:**

This case report highlights how a novel liquid biopsy diagnostic test based on the detection of exosome protein biomarkers allowed early diagnosis of a high-grade precancerous lesion for PDAC and improved patient outcome.

## 1 Introduction

Pancreatic cancer is a deadly disease that is expected to become the second-leading cause of cancer-related death in the United States by 2030 (1). The poor prognosis of pancreatic cancer is, in part, a by-product of current methods for diagnosing pancreatic ductal carcinoma (PDAC) failing to identify early stage (localized) cases. Most cases (76.6%) are diagnosed at later stages (regional or distant) when the window for curative surgical intervention has closed (2). Despite these late confirmatory diagnoses, there is increasing evidence that patients present with symptoms of cancer before metastasis, but these symptoms can be inadvertently overlooked due to their vague nature or patients themselves lose motivation to undergo imaging (3).

Currently, carbohydrate antigen 19-9 (CA19-9) is the only FDA-approved and most widely used biomarker for pancreatic cancer (4). However, CA19-9 is mainly limited to patient surveillance and follow-up since it lacks the necessary sensitivity and specificity as an early detection diagnostic marker (5, 6). In addition, CA19-9 can be falsely elevated in certain benign conditions, such as pancreatitis, as well as in other gastrointestinal malignancies, which further limits its use as a clinically reliable biomarker (7).

Alternatively, most current liquid biopsy tests rely on sequencing and detection of cancer-derived fractions of cell-free DNA (cfDNA) (8). Since many of these tests have been developed for screening *average*-*risk* patients, they face challenges for use with high-risk patients. As an example, the commercially-available test, Galleri^®^ by Grail, has a 16.8% sensitivity for stage I pan-cancer, which severely limits the test’s ability to rule out the presence of cancer (9). Furthermore, low sensitivity tests are not appropriate when diagnosing patients for the presence of cancer that are at high-risk due to symptoms or other clinical factors.

We recently developed a laboratory developed test (LDT), ExoVita^®^ by Biological Dynamics, which isolates exosomes from EDTA-anticoagulated blood for the classification of PDAC cases and have optimized our performance for sensitivity for high-risk individuals. This test was further evaluated in a case-control study where pathologically-confirmed stage I and II PDAC patients were identified with 96% sensitivity at 91.1% specificity, suggesting that this test could fill a diagnostic gap for managing high risk patients in conjunction with other standard-of-care (SOC) modalities (10). The test methodology is described in the Supplementary Material.

Here, we present the case of a patient with acute pancreatitis who underwent a diagnostic workup that included both SOC and novel biomarker assays. While SOC diagnostic tests, including CA 19-9 and CEA, as well as novel cell-free DNA testing and imaging modalities failed to identify cancerous lesions in this patient, the ExoVita test by Biological Dynamics indicated a high probability of PDAC. Appropriate follow-up testing and surgical intervention led to symptom resolution and improved patient outcome.

## 2 Case description

**Figure 1:**
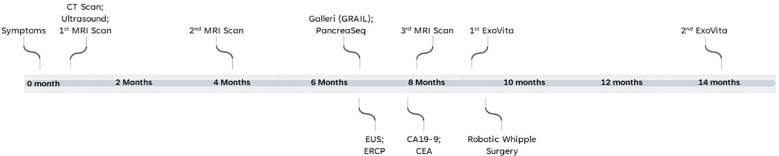
Timeline and events of patient’s clinical journey.

A 60-year-old healthy non-Hispanic white male with no previous medical or family history presented to clinic in January 2022 with persistent symptoms of abdominal bloating and pressure for four to five days. An initial abdominal computed tomography (CT) scan led to a diagnosis of acute pancreatitis. After symptom management, ultrasound (US) and magnetic resonance imaging (MRI) of the abdomen one-week after the episode showed an abnormal dilatation of the main pancreatic duct. The MRI was repeated after 4 months and it showed that the duct dilation was unchanged. Timeline of events is report in **Figure 1**. The main findings of the imaging and clinical biomarker tests are reported in **Supplementary Table 1**.

## 3 Diagnostic assessment

Due to persistent pancreatic duct dilatation in the context of normal post-acute pancreatitis course, an endoscopic ultrasound (EUS) was later performed that showed an abrupt stricture of the pancreatic duct with no evidence of masses or lesions. Two weeks after the EUS procedure, an endoscopic retrograde cholangiopancreatography (ERCP) was performed with stent placement due to complete ductal occlusion. Cytology brushings taken at the time of the ERCP were non-diagnostic. Having been educated about diagnostics and genomics, the patient chose to have the cytology brushings tested with tissue-based 22-gene panel (PancreaSeq^®^, University of Pittsburgh Medical Center) to explore if there were any underlying genetic alterations (11). The results of the DNA-sequencing revealed the presence of KRAS mutation p.G12D, c.35G>A at 11% median variant allele frequency (VAF), TP53 mutation p.Y205F, c.614A>T at 15% median VAF, and copy number alterations of RNF43, increasing the clinical concern.

In the two months following the sequencing results, the patient elected for several additional biomarker analyses. The patient had a multi-cancer early detection (MCED) test analyzing cfDNA (Galleri by GRAIL), measurement of serum CA-19-9 and CEA protein levels, an additional MRI, and the ExoVita test, a liquid biopsy LDT that analyzes proteins from plasma-derived exosomes (10).

Neither the Galleri test, which reported “Cancer Signal Not Detected”, nor CA-19-9 and CEA, which were non-elevated (CA19-9 = 3.0 U/ml, CEA = 3 ng/ml), raised disease concerns. However, the ExoVita test indicated a high probability of PDAC.

**Figure 2:**
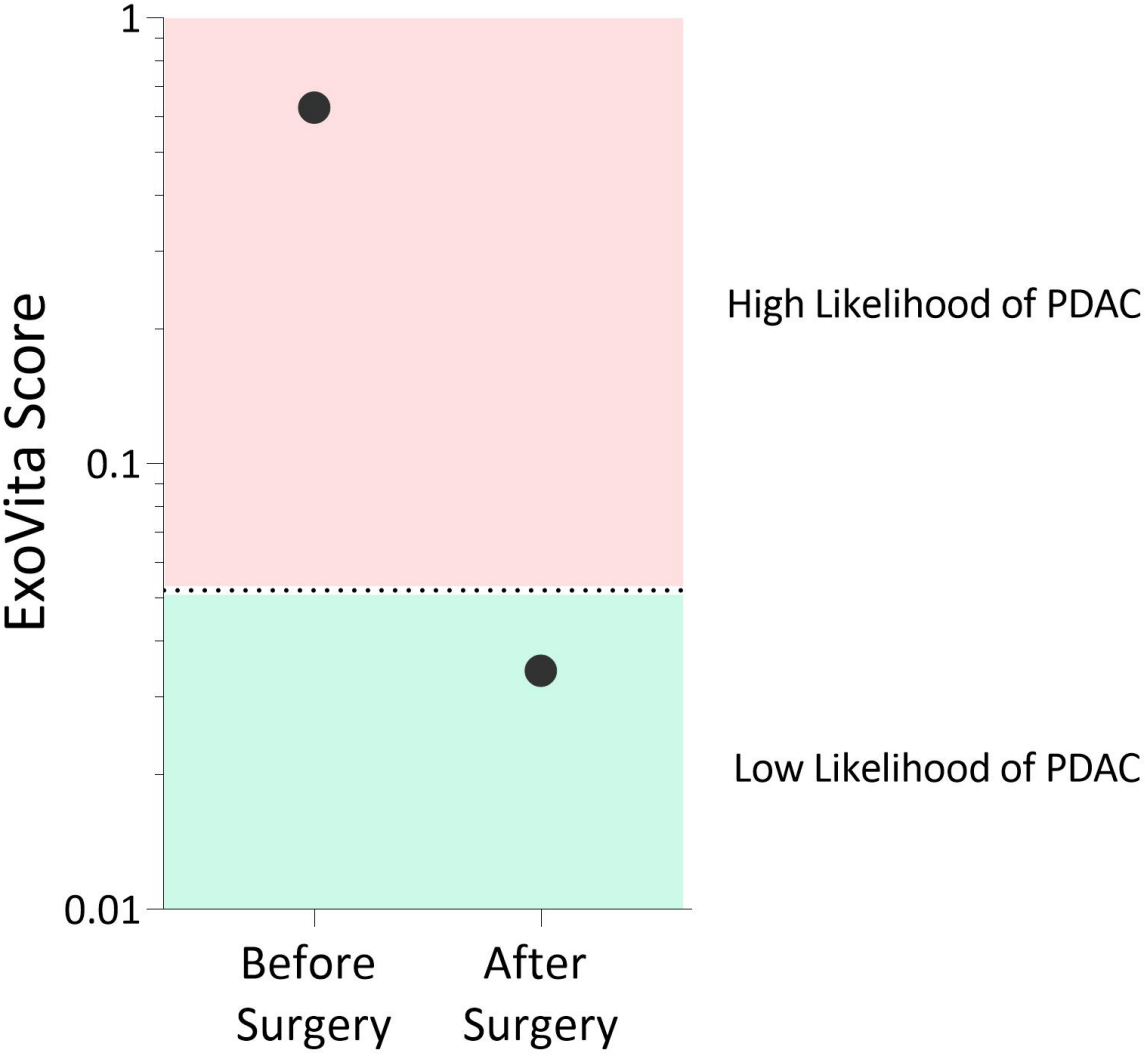
Results of the ExoVita test 10 days before and 5 months after pancreaticoduodenectomy.

Although the MCED test, as well as CA-19-9 and CEA, were not indicative of cancer in this patient, this is not surprising given that these biomarkers have suboptimal sensitivity and, thus, a relatively high percentage of cases are false negatives (6, 9). Conversely, the ExoVita test has shown high sensitivity (12) for PDAC at stages 1 and 2. The ExoVita test, combined with the presence of germline mutations in KRAS and TP53 genes, indicated high risk of developing cancer in this patient. After careful consideration of the test results and multiple differing surgical opinions, the patient elected to undergo a robotic pancreaticoduodenectomy (Whipple) procedure. Final pathology of the resected pancreatic tissue revealed the presence of an intraductal papillary mucinous neoplasm (IPMN) of pancreatobiliary type with focal high-grade dysplasia primarily involving the main duct (MD-IPMN) with extension into a branch duct (BD-IPMN) in addition to high-grade PanIN, none of which had not been detected by previous multiple imaging modalities. The patient’s post-operative course was unremarkable. At five months status post Whipple procedure, the patient continues to recover well without complications. In addition, a repeat of the ExoVita test indicated low probability of cancer with all biomarkers expressed from exosomes appropriately declined. The patient’s two ExoVita results are reported in **Figure 2**.

## 4 Discussion

This case report illustrates the clinical utility of using a novel biomarker test such as the ExoVita test to categorize the probability of pre-cancerous lesions that were missed despite an extensive workup using multiple imaging modalities (See **Figure 1** for timeline and events of patient’s clinical journey and **Supplementary Table 1** for main test results). Precancerous lesions place patients at high-risk of progression onto pancreatic cancer. According to the revised Fukuoka guidelines (13) for the management of IPMNs, the mean frequency of developing invasive carcinoma and high-grade dysplasia in patients with worrisome main duct IPMN is 61.6% (range, 36 - 100%) and the recommendation is surgical resection of the pancreas. Specifically, there is wide consensus among most clinicians that all MD-IPMN and mixed-type IPMNs should be treated as potentially malignant lesions and surgical resection is advised. The prognosis for IPMN after surgery is generally favorable and a recent large study by Griffin, et al. (14) concluded that post-surgical IPMN patients had a 1-year survival rate of 92.7%, and the 5-year survival rate was 72.9% in contrast with late stage cancer diagnoses, which are 13% and 3% for stages II/III and IV, respectively (14, 15). Patients with surgically resected non-invasive benign IPMN have an even better prognosis, with 10-year disease-specific survival rates of >95% for both MD- and BD-IPMN (16).

This case study highlights how by combining a high-sensitivity biomarker panel with imaging-based modalities, high-risk patients may achieve a faster and more cost-effective diagnosis allowing for informed treatment decisions. Although more studies are required to assess patient outcomes, we speculate that early use of ExoVita test in patients suspected of PDAC (either before or concurrently with initial imaging), could mitigate delays and costs from additional testing. Current screening based MCED tests harbor impressive specificity values (9). However, sensitivity for stage I cancers remains far from ideal (17), limiting their ability for use in high-risk patients. The patient described in this case report received one of these tests during his workup; however, that test failed to detect the presence of cancer, and arguably may have delayed curative surgery and increased the overall cost of his diagnostic journey. Recently, it has been suggested that high-sensitivity screening tests should be the preferred approach over high-specificity to enrich for disease populations (18).

SOC biomarkers for pancreatic cancer include elevated serum levels of CA19-9 and CEA, with the former considered a worrisome feature for the presence of high-grade dysplasia or invasive carcinoma based on current guidelines (13). However, sensitivity of CA19-9 in malignant and invasive IPMN is low (40-52%), indicating that many patients have these conditions despite normal serum levels of CA19-9 (19). In addition, CA19-9 can be elevated in patients with benign diseases (peptic ulcers, pancreatitis, cirrhosis, cholangitis, and obstructive jaundice) (19), making the differential diagnosis challenging. Also, CEA has low sensitivity, and, thus, it is unsuitable as a ‘screening’ method for malignant and invasive IPMN, especially in high-risk patients. However, specificity of CEA can be generally >90%, allowing it to be used to rule-in an IPMN malignancy (19). Non-invasive liquid biopsy tests have been recently developed for the diagnosis and prognosis of cancer, including pancreatic cancer. These tests measure circulating tumor DNA (ctDNA), which is released into the plasma by necrotic tumor cells; circulating tumor cells (CTCs), which derive from primary cancer, are released into the lymphatics or vasculature, and are carried throughout the body; or exosomes, sub-nanoparticles of extracellular vesicles that mediate cell-to-cell communication and carry cargo such as proteome, DNA, RNA, and other metabolites. A recent meta-analysis showed that the exosome-based assays have the highest sensitivity, specificity, and area under the ROC curve (20).

The patient described in this case report received SOC and one of the newly developed liquid biopsy tests (the MCED test, also called Galleri by GRAIL), which uses cfDNA sequencing, during his workup. However, these tests failed to detect the presence of cancer, and arguably may have delayed curative surgery and increased the overall cost of his diagnostic journey. Recently, it has been suggested that high-sensitivity screening tests should be the preferred approach over high-specificity to enrich for disease populations (18).

Liquid-biopsy tests require a simple blood draw and thus, offer the opportunity to increase access and patient adherence compared to imaging methods, which require an outpatient visit. We hypothesize that incorporation of a test like the ExoVita test into surveillance and/or diagnostic programs for PDAC high-risk patients will be a cost-effective way to address an unmet need in the community setting by elevating patients with a high probability of cancer to more intensive management programs, as well as stage-shift patients to early-stage diagnoses. Further investigations on this topic are underway including a prospective, multi-center, observational registry study – ExoLuminate – to evaluate patients at high-risk for PDAC (NCT0562552) (21) as well as health economic and cost-effectiveness utility studies. In conclusion, this case report highlights how a novel diagnostic test based on the detection of exosomes biomarkers allowed early diagnosis of PDAC and improved patient outcome.

## Data availability statement

The original contributions presented in the study are included in the article/**Supplementary Material**. Further inquiries can be directed to the corresponding author.

## Ethics statement

The studies involving human participants were reviewed and approved by Western Institutional Review. Board-Copernicus Groupо (WCGIRBо) under study number 1155036, sub-nanoparticles of extracellular vesicles that mediate cell-to-cell communication and carry cargo such as proteome, DNA, RNA, and other metabolites. IRB tracking number 20150581. Written informed consent was obtained from the individual for the publication of any potentially identifiable images or data included in this article.

## Author contributions

All authors listed have made a substantial, direct, and intellectual contribution to the work and approved it for publication.

## References

[1] Rahib L Wehner MR Matrisian LM Nead KT. Estimated projection of US cancer incidence and death to 2040. JAMA Network Open (2021) 4(4):e214708–e. doi: 10.1001/jamanetworkopen.2021.4708 PMC8027914 33825840

[2] Lennon AM Wolfgang CL Canto MI Klein AP Herman JM Goggins M. The early detection of pancreatic cancer: what will it take to diagnose and treat curable pancreatic neoplasia? Cancer Res (2014) 74(13):3381–9. doi: 10.1158/0008-5472.CAN-14-0734 PMC4085574 24924775

[3] Grossberg AJ Chu LC Deig CR Fishman EK Hwang WL Maitra A. Multidisciplinary standards of care and recent progress in pancreatic ductal adenocarcinoma. CA: A Cancer J Clin (2020) 70(5):375–403. doi: 10.3322/caac.21626 PMC7722002 32683683

[4] Kane LE Mellotte GS Conlon KC Ryan BM Maher SG. Multi-omic biomarkers as potential tools for the characterisation of pancreatic cystic lesions and cancer: innovative patient data integration. Cancers (2021) 13(4):769–91. doi: 10.3390/cancers13040769 PMC7918773 33673153

[5] Le N Sund M Vinci A Beyer G Ashan Javed M Krug S. Prognostic and predictive markers in pancreatic adenocarcinoma. Digestive Liver Disease (2016) 48(3):223–30. doi: 10.1016/j.dld.2015.11.001 26769569

[6] Swords DS Firpo MA Scaife CL Mulvihill SJ. Biomarkers in pancreatic adenocarcinoma: current perspectives. Onco Targets Ther (2016) 9:7459–67. doi: 10.2147/OTT.S100510 PMC5158171 28003762

[7] Luo G Fan Z Cheng H Jin K Guo M Lu Y. New observations on the utility of CA19-9 as a biomarker in Lewis negative patients with pancreatic cancer. Pancreatology (2018) 18(8):971–6. doi: 10.1016/j.pan.2018.08.003 30131287

[8] Hackshaw A Clarke CA Hartman A-R. New genomic technologies for multi-cancer early detection: rethinking the scope of cancer screening. Cancer Cell (2022) 40(2):109–13. doi: 10.1016/j.ccell.2022.01.012 35120599

[9] Klein EA Richards D Cohn A Tummala M Lapham R Cosgrove D. Clinical validation of a targeted methylation-based multi-cancer early detection test using an independent validation set. Ann Oncol (2021) 32(9):1167–77. doi: 10.1016/j.annonc.2021.05.806 34176681

[10] Sears R Hinestrosa J Schroeder G Lewis J Balcer H Kurzrock R. 1306P early-stage pancreatic cancer detection using extracellular vesicles. Ann Oncol (2022) 33:S1141. doi: 10.1016/j.annonc.2022.07.1438

[11] Paniccia A Polanco PM Boone BA Wald AI McGrath K Brand RE. Prospective, multi-institutional, real-time next-generation sequencing of pancreatic cyst fluid reveals diverse genomic alterations that improve the clinical management of pancreatic cysts. Gastroenterology (2022) 164(1):117–33.e7. doi: 10.1053/j.gastro.2022.09.028 PMC9844531 36209796

[12] Hinestrosa JP Kurzrock R Lewis JM Schork NJ Schroeder G Kamat AM. Early-stage multi-cancer detection using an extracellular vesicle protein-based blood test. Commun Med (2022) 2(1):29. doi: 10.1038/s43856-022-00088-6 35603292 PMC9053211

[13] Tanaka M Fernández-Del Castillo C Kamisawa T Jang JY Levy P Ohtsuka T. Revisions of international consensus Fukuoka guidelines for the management of IPMN of the pancreas. Pancreatology (2017) 17(5):738–53. doi: 10.1016/j.pan.2017.07.007 28735806

[14] Griffin JF Page AJ Samaha GJ Christopher A Bhaijee F Pezhouh MK. Patients with a resected pancreatic mucinous cystic neoplasm have a better prognosis than patients with an intraductal papillary mucinous neoplasm: a large single institution series. Pancreatology (2017) 17(3):490–6. doi: 10.1016/j.pan.2017.04.003 28416122

[15] American Cancer Society. Cancer facts & figures 2022. American Cancer Society (2022).

[16] D'Haese JG Werner J. Surgery of cystic tumors of the pancreas - why, when, and how? Visc Med (2018) 34(3):206–10. doi: 10.1159/000489234 PMC6117660 30182025

[17] Pons-Belda OD Fernandez-Uriarte A Diamandis EP. Can circulating tumor DNA support a successful screening test for early cancer detection? Grail Paradigm. Diagnostics (Basel) (2021) 11(12):2171–8. doi: 10.3390/diagnostics11122171 PMC8700281 34943407

[18] Fiala C Diamandis EP. A multi-cancer detection test: focus on the positive predictive value. Ann Oncol (2020) 31(9):1267–8. doi: 10.1016/j.annonc.2020.05.028 32741675

[19] Nista EC Schepis T Candelli M Giuli L Pignataro G Franceschi F. Humoral predictors of malignancy in IPMN: a review of the literature. Int J Mol Sci (2021) 22(23):12839. doi: 10.3390/ijms222312839 34884643 PMC8657857

[20] Zhu Y Zhang H Chen N Hao J Jin H Ma X. Diagnostic value of various liquid biopsy methods for pancreatic cancer: a systematic review and meta-analysis. Med (Baltimore). (2020) 99(3):e18581. doi: 10.1097/MD.0000000000018581 PMC7220382 32011436

[21] ExoLuminate study for early detection of pancreatic cancer. Available at: https://ClinicalTrials.gov/show/NCT05625529.

