# Supplementary material for: Case Report: Early detection of pancreatic pre-cancer lesion in multimodal approach with exosome liquid biopsy
Source: Front Oncol. 2023 May 11;13:1170513. doi: 10.3389/fonc.2023.1170513 (PMC10210587; doi:10.3389/fonc.2023.1170513)
Supplement: Supplementary file 1 [file DataSheet_1.docx]

**Case Report: Early Detection of Pancreatic Pre-cancer Lesion in Multimodal Approach with Exosome Liquid Biopsy**

**SUPPLEMENTARY MATERIAL**

The ExoVita test was performed at Biological Dynamics as previously described (1). Briefly, EDTA-anticoagulated blood was centrifuged twice to isolate plasma and 280 µL of undiluted plasma was introduced into an alternating current electrockinetics (ACE) platform (Verita™ chip from Biological Dynamics). An electrical current of 7 Vpp and 14 KHz was applied while plasma moved across the chip at 3 µL/min for 120 min. These conditions allow exosomes to be captured onto the energized microelectrode array. Following a wash to eliminate unbound particles, the exosomes are eluted from the cartridge with a proprietary elution buffer. Three different bead-based immunoassay kits (Human Circulating Biomarker Magnetic Bead Panel 1 (Cat # HCCBP1MAG-58K), Human Angiogenesis Magnetic Bead Panel 2 (Cat # HANG2MAG-12K), and Human Circulating Cancer Biomarker Panel 3 (Cat # HCCBP3MAG58K), all from Millipore Sigma (Burlington, MA) were used for downstream biomarker analysis using 25 µL of purified exosomes per kit. Multiplex ELISA were performed according to the manufacturer’s protocols using the MAGPIX system (Luminex Corp, Austin, TX). Belysa software v. 3.0 (EMD Millipore) was used to determine the protein concentrations from calibration curves. Biomarker selection and determination of the coefficients of each biomarker in the logistic regression were described previously (2). Finally, a proprietary algorithm provided a score for probability of pancreatic cancer.

1. Sears R, Hinestrosa J, Schroeder G, Lewis J, Balcer H, Kurzrock R, et al. 1306P Early-stage pancreatic cancer detection using extracellular vesicles. Annals of Oncology. 2022;33:S1141.

2. Hinestrosa JP, Kurzrock R, Lewis JM, Schork NJ, Schroeder G, Kamat AM, et al. Early-stage multi-cancer detection using an extracellular vesicle protein-based blood test. Communications Medicine. 2022;2(1):29.

**Supplementary Table 1. Main findings of imaging and clinical biomarker tests.**

| **Imaging, Laboratory Tests, and Biomarkers** | **Date** | **Results** |
| --- | --- | --- |
| **CT scan** | Jan 2022 | - Diffuse MD dilation up to 6 mm |
| **Serum Lipase** | Jan 2022 | - Normal |
| **Ultrasound (US)** | Jan 2022 | - MD dilation |
| **MRI** | Jan 2022 | - MD dilation (6 mm) - No pancreatic mass |
| **MRI** | May 2022 | - Stable MD dilation (6 mm). - No pancreatic mass. |
| **Endoscopic ultrasound (EUS) and Endoscopic retrograde cholangiopancreatography (ERCP)** | Aug 2022 | - MD stricture (8 mm long). - Stent placement. - No pancreatic mass. - Few pancreatic cysts (2-6 mm). - No high risk or worrisome features. |
| **Cytology brushings (22-gene panel: PancreaSeq®)** | Aug 2022 | - KRAS mutation p.G12D, c.35G>A at 11% median variant allele frequency (VAF) - TP53 mutation p.Y205F, c.614A>T at 15% VAF - RNF43 copy number alterations |
| **Multi-cancer early detection (MCED) liquid biopsy test (cell-free DNA: Galleri® by GRAIL)** | Aug 2022 | - Cancer Signal Not Detected |
| **MRI** | Sep 2022 | - Stable MD dilation (5 mm) - Dilated branch ducts - MD stricture (3mm long) - No ductal calculus - No ductal wall thickening or enhancement |
| **CA19-9** | Sep 2022 | - 3 U/ml (normal) |
| **CEA** | Sep 2022 | - 3 ng/ml (normal) |
| **EV-Based Test** | Oct 2022 | - High probability of PDAC |
| **Robotic Whipple (Pancreatoduodenectomy) Surgery in October 2022** | | |
| **Pathology of the resected pancreatic tissue during robotic Whipple Surgery** | Oct 2022 | - Intraductal papillary mucinous neoplasm (IPMN) of pancreatobiliary type. - Focal high-grade dysplasia primarily involving the main duct (MD-IPMN) with extension into a branch duct (BD-IPMN) - High-grade PanIN - Regional lymph nodes: negative |
| **EV-Based Test** | Mar 2023 | - Low probability of PDAC |

MD: main pancreatic duct; MRI: magnetic resonance imaging EV: extracellular vesicles.
